# Vitamin D Deficiency and Its Associated Factors among Female Migrants in the United Arab Emirates

**DOI:** 10.3390/nu14051074

**Published:** 2022-03-03

**Authors:** Fatme Al Anouti, Luai A. Ahmed, Azmat Riaz, William B. Grant, Nadir Shah, Raghib Ali, Juma Alkaabi, Syed M. Shah

**Affiliations:** 1 Department of Health Sciences, College of Natural and Health Sciences, Zayed University, Abu Dhabi 144534, United Arab Emirates; fatme.alanouti@zu.ac.ae; 2 Institute of Public Health, College of Medicine and Health Sciences, United Arab Emirates University, Al Ain 17666, United Arab Emirates; luai.ahmed@uaeu.ac.ae; 3 Department of Obstetrics and Gynecology, Ajman University, Ajman 20550, United Arab Emirates; azmatriazkhan@gmail.com; 4 Sunlight, Nutrition and Health Research Center, P.O. Box 641603, San Francisco, CA 94164-1603, USA; wbgrant@infionline.net; 5 Planning and Development Division, Government of Gilgit Baltistan, Gilgit 15100, Pakistan; nadir.shah.sun@gmail.com; 6 Public Health Research Center, New York University Abu Dhabi, Abu Dhabi 129188, United Arab Emirates; ra107@nyu.edu; 7 Department of Internal Medicine, College of Medicine and Health Sciences, United Arab Emirates University, Al Ain 17666, United Arab Emirates; j.kaabi@uaeu.ac.ae; 8 Department of Family Medicine, Aga Khan University, Karachi 3500, Pakistan

**Keywords:** vitamin D status, prevalence, female migrants, United Arab Emirates

## Abstract

Vitamin D is important for bone health, and vitamin D deficiency could be linked to noncommunicable diseases, including cardiovascular disease. The purpose of this study was to determine the prevalence of vitamin D deficiency and its associated risk factors among female migrants from Philippines, Arab, and South Asian countries residing in the United Arab Emirates (UAE). We used a cross-sectional study to recruit a random sample (*N* = 550) of female migrants aged 18 years and over in the city of Al Ain, UAE. Vitamin D deficiency was defined as serum 25-hydroxyvitamin D concentrations ≤20 ng/mL (50 nmol/L). We used multivariable logistic regression analysis to identify risk factors associated with vitamin D deficiency. The mean age of participants was 35 years (SD ± 10). The overall prevalence rate of vitamin D deficiency was 67% (95% CI 60–73%), with the highest rate seen in Arabs (87%), followed by South Asians (83%) and the lowest in Filipinas (15%). Multivariate analyses showed that low physical activity (adjusted odds ratio (aOR) = 4.59; 95% CI 1.98, 10.63), having more than 5 years duration of residence in the UAE (aOR = 4.65; 95% CI: 1.31, 16.53) and being obese (aOR = 3.56; 95% CI 1.04, 12.20) were independently associated with vitamin D deficiency, after controlling for age and nationality. In summary, vitamin D deficiency was highly prevalent among female migrants, especially Arabs and South Asians. It is crucial that health professionals in the UAE become aware of this situation among this vulnerable subpopulation and provide intervention strategies aiming to rectify vitamin D deficiency by focusing more on sun exposure, physical activity, and supplementation.

## 1. Introduction

Vitamin D deficiency remains a worldwide public health problem, affecting large proportions of the population in developed and the developing countries [1]. Vitamin D deficiency remains common in children and adults and the musculoskeletal consequences of inadequate vitamin D are well established. In utero and during childhood, vitamin D deficiency can lead to growth retardation and skeletal deformities including childhood rickets [2]. Vitamin D supplementation during pregnancy is associated with improved infant growth and reduction of fetal or neonatal mortality [3]. In adults, vitamin D deficiency can cause osteomalacia, muscle weakness and increased risk of fracture [4]. Although the strongest evidence for the effect of vitamin D deficiency is related to skeletal disorders, low concentrations of vitamin D are associated with several non-skeletal disorders including cardiovascular diseases, several types of cancer, neurodegenerative diseases, disorders of glucose metabolism, and a possible role in the recently emerging pandemic of COVID-19 [5,6,7,8].

The extreme consequences of vitamin D deficiency, such as rickets in children and osteomalacia in adults, have been almost eliminated in some developed countries through adequate diet, food fortification, and the encouragement of moderate sunlight exposure [9]. Paradoxically, populations in the Middle East and North Africa (MENA) region have some of the lowest serum 25-hydroxy-vitamin D [25(OH)D] concentrations worldwide, despite the abundance of sunshine throughout the year [10,11].

Vitamin D deficiency among women in Arab countries has been attributed to inadequate exposure of skin to sunlight due to a very conservative style of dress that covers most of the body when they are outdoors [11,12]. In a study of Arab-American women, there was a significantly higher prevalence of vitamin D deficiency in women practicing a conservative style of dress, compared to their counterparts practicing a less conservative dressing [13,14]. We previously examined the prevalence of vitamin D deficiency among adolescents aged 15 to 18 years in the city of Al Ain, UAE. A higher proportion (32.0%) of female adolescents had vitamin D deficiency as compared to their male counterparts (8.0%) [15].

Given the remarkably high percentage of working migrants, known as ‘guest workers’ or ‘expatriates’, the UAE is one of the most culturally diverse countries. Expatriate foreign workers account for almost 80% of the UAE’s total population [16]. A significant proportion of female migrant workers, including women from various Arab countries, South Asia, and Philippines are typically employed for indoor work activities, such as domestic or office work, sales, and beauty salons.

Migrant workers are among the most vulnerable populations that could be afflicted with vitamin D deficiency [17]. This study aimed to estimate the prevalence of vitamin D deficiency and examine the correlates of low levels of serum 25(OH)D concentrations among Arab, South Asian, and Filipina migrants residing in the UAE. To our knowledge this is the first research investigation that targets this under-studied subpopulation to assess the burden of vitamin D deficiency and pave for future studies that could aim to address such an important public health issue.

## 2. Methods

### 2.1. Study Design and Ethics

The study employed a cross-sectional design. We obtained a College of Medicine and Health Sciences, UAE individual faculty grant for the project, entitled “Chronic Diseases Prevention in Immigrants: putting CVD risk factors on surveillance screen”. Ethical approval was obtained from the Al Ain Medical District Human Research (AAMDHREC 10/21) and study participants provided written informed consent.

### 2.2. Selection of Study Participants

The target study population consisted of female Arab, South Asian, and Filipina migrant workers aged 18 years and older. We used the formula for binomial distribution (*n*= ^z^zα^2^ p (1 − p)/d^2^) to estimate the sample size, where (n) is the sample size, (zα) is the normal deviate (1.96) at 5% level of significance, (p) is the prevalence, and (d) is the precision. Assuming a precision or tolerable variation of ±0.06 around an estimated prevalence of 70% for vitamin D deficiency in women [9], a sample of 200 participants would be needed.

All expatriate workers seeking employment or renewing their visa in the UAE are required by law to undergo health and communicable disease screening. The sampling frame in this study was a list of all expatriate workers from the Arab region, South Asia, and Philippines who were enrolled for medical examination at the only visa screening center in the city of Al Ain, Abu Dhabi emirate, during the process of either obtaining a new visa or renewing it over a period of six months [18].

### 2.3. Inclusion Criteria

Female migrant workers who were of an Arab, South Asian, or Philippines’ nationality, aged ≥18 years, were able to read and speak Arabic, Urdu, Hindi, Bengali, or Filipino, and to provide a written informed consent were eligible to participate in the study. Due to the low literacy rate among the South Asian expatriate population in the UAE [16], the study questionnaire was interviewer-administered, and all interviews were conducted in Urdu, Hindi, Bengali, or Filipino, and led by native Urdu, Hindi, Bengali or Filipino speaking research assistants who had received appropriate training. Eligible participants registered for the visa screening were invited to participate in the study which spanned over six months to complete recruitment of the required sample.

### 2.4. Measures

We used an adapted version of the World Health Organization (WHO) global standard “STEPS” survey questionnaire entitled “Chronic Diseases Prevention in Immigrants: putting CVD risk factors on surveillance screen” for population-based assessment of the prevalence of noncommunicable diseases (NCDs) risk factors including vitamin D deficiency [19].

A 5 mL venous fasting blood sample was obtained from the study participants by qualified nurses using standardized tubes. Blood samples were immediately transferred to Tawam Hospital (Al Ain) laboratory where they underwent standardized (quality controlled) analyses. Serum 25(OH)D concentrations were measured by radioimmunoassay (DiaSorin, Stillwater, Minnesota, MN, USA). The intra-assay and inter-assay for coefficients of variation were 8.3% and 3.2% respectively. We used the reference value for serum 25(OH)D concentrations <20 ng/mL (50 nmol/L) to define vitamin D deficiency [4].

We collected information on demographics, lifestyle factors, family and personal disease history, home country residence setting (rural, urban), occupation, and monthly salary in UAE dirham or AED (USD1.00 ~ AED3.67).

Studies in migrants from Western developed countries indicated a decline in health with the increased duration of stay, which could be attributed to the adoption of local behaviors and norms and diet, also known as acculturation [20]. We used the duration of residence as a marker to evaluate the effect of acculturation on vitamin D status among the participants [21].

Participants’ weight and height measurements were performed using standard weight and height scales (SECA, Hamburg, Germany). Body mass index (BMI) was calculated as weight in kilograms divided by the square of the height in meters and BMI categorization was based on WHO recommendations: being overweight (BMI 25.0 to 29.9 kg/m^2^), and obesity (≥30.0 kg/m^2^) [22]. Resting brachial blood pressure (BP) was measured using a calibrated automated BP measurement device (Omron HEM-705cp) in sitting position using the right upper arm and an appropriately sized cuff after a period of five minutes’ rest. The average of two measures taken was used for analysis. Hypertension (HTN) was defined as being on anti-hypertensive medications or having a systolic blood pressure ≥140 mmHg or a diastolic blood pressure ≥90 mmHg [23]. Study participants were classified as current smokers if they answered yes to the question, *“*have you ever smoked cigarettes, cigars or shisha?”. Information on physical activity was obtained using the International Physical Activity Questionnaire (IPAQ-short version) [24]. We measured the frequency (days per week) and duration (minutes per day) of moderate- and vigorous-intensity physical activity in a period of seven-days prior to the survey. Physical activity was based on recall of daily activity patterns in the previous 7 days. Using the US guideline for physical activity, recommended by the Centers for Disease Control and Prevention (CDC) and the American College of Sports Medicine (ACSM), we identified the proportion of participants reporting moderate-intensity physical activity for a minimum of 30 min on five days each week or vigorous-intensity physical activity for a minimum of 20 min on three days each week [25].

### 2.5. Statistical Analysis

Data were analyzed using SPSS version 27.0 (IBM, Armonk, NY, USA). Categorical variables were presented as frequencies and percentages. Continuous variables were presented as mean ± standard deviation. Participants were grouped into three categories based on serum 25(OH)D levels and all variables were compared using Chi-Square test for categorical variables and One-Way Analysis of Variance (ANOVA) for continuous variables. Moreover, differences in results among the participants (Arabs, Asians, Filipinas) were explored using Chi-Square test for categorical variables and Independent-Samples *t*-Test for continuous variables. Simple and multivariate ordered logistic regression models were constructed to determine the predictors of vitamin D deficiency. Simple and multivariate binary logistic regression models were considered to identify the variables associated with vitamin D deficiency. A *p*-value of <0.05 was considered statistically significant in our analyses.

## 3. Results

The mean age of study participants was 35 years (SD ± 10), 33 years (SD ± 8) for Filipinas, 37 years (SD ± 11) for Arabs, and 34 years (SD ± 10) for South Asian. Table 1 shows the characteristics of the study population.

A significant proportion (7.2%) had no formal schooling, 37.2% had secondary level education and 55.6% had college or higher levels of education. A high proportion (57.4%) were married, 36.4% were unmarried and 6.2% were divorced or widowed. A high proportion of study participants worked as housemaids (36.6%) and housewives (23.3%). The rest were health care workers (10.5%), teachers (6.9%), administrators or supervisors (6.7%), drivers or cooks (4%), and multitude of other activities (10.5%). With regards to background in home country, 52.8% were from urban settings while 47.2% had rural backgrounds. The prevalence of obesity in Filipinas was notably low (6.9%) as compared to their Arabs (37.5%) and South Asians (17.3%) counterparts. Moderate and vigorous physical activity was reported by a higher proportion of Filipinas (45.9%) as compared to Arab (11.0%) and South Asian (10.2%) female immigrants.

Table 2 shows the mean ± SD of 25(OH)D concentrations and vitamin D deficiency in the study population.

Overall, the 25(OH)D concentrations were 20 ± 11 ng/mL in the study population, specifically 30 ± 11 in the Filipinas, 14 ± 10 in Arabs, and 15 ± 9 in South Asians. The overall prevalence rate of vitamin D deficiency (25(OH)D ≤ 20 ng/mL) was 66.7% (95% CI, 60.0%–72.7%). Examination of the prevalence rate of vitamin D deficiency by age revealed insignificant variations. There were significant differences in 25(OH)D concentrations as well as the prevalence rate of vitamin D deficiency by nationality, education level, occupation, income, duration of residence in the UAE, physical activity, body mass index categories, and self-reported alcohol use. There was an inverse correlation between earnings and vitamin D status and similarly an inverse correlation between a high education level and lower vitamin D status. This was due to the fact that vitamin D deficiency is much lower (15.8%) when compared to Arab (86.7%) and South Asian (83.3), as shown in Table 1. A high proportion of Filipinas (52.5%) were in the lowest tertile of monthly earning when compared to Arab (13.2%) and South Asian (6.5%) in the lowest tertile of monthly earning. Similarly, a high proportion of Arab and South Asian had college or higher level of education. A high proportion of Filipinas reported alcohol consumption (33.4%) compared to their (Arab (0.7%) and South Asian (5.55) counterparts.

**Figure 1:**
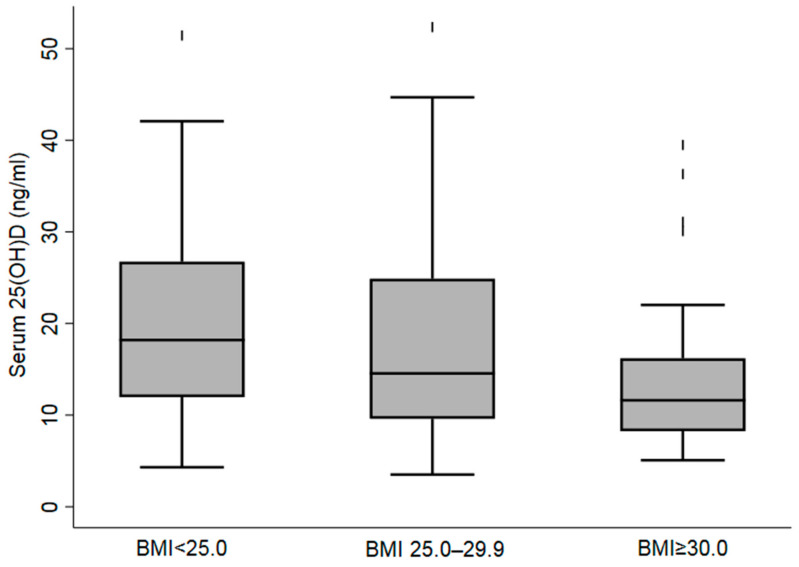
Boxplot of Serum 25(OH)D Concentrations according to Body Mass Index in the Female Migrant Participants Living in Al Ain, UAE.

**Figure 2:**
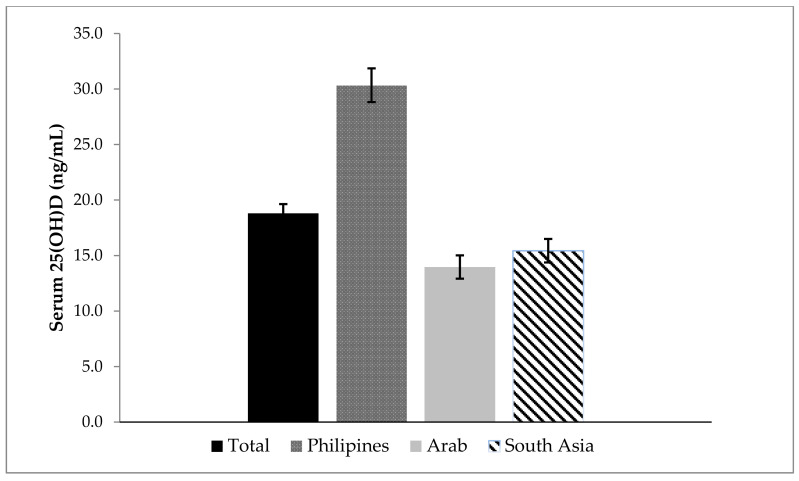
Mean Serum 25(OH)D Concentrations ± SEM in the Females Migrant Participants Living in Al Ain, UAE.

The boxplot of participants’ serum 25(OH)D concentrations (ng/mL) according to BMI categories in Figure 1 demonstrates the low levels of 25(OH)D concentrations (ng/dL) among overweight and obese participants. Figure 2 shows participants’ 25(OH)D concentrations levels overall, and by the nationality.

Table 3 summarizes the results from multivariate analyses. After adjusting for all related factors, including age and nationality, low physical activity (AOR = 4.59; 95% CI 1.1.98–10.63), having more than 5 years duration of residence in UAE (AOR = 4.65; 95% CI 1.31–16.53), and being obese (AOR = 3.56; 95% CI 1.04–12.20) were independently associated with vitamin D deficiency.

## 4. Discussion

This study showed the extent of vitamin D deficiency among female migrants. Vitamin D deficiency was especially common among Arab and South Asian migrants. Using serum 25(OH)D concentrations ≤20 ng/mL as a cutoff to define vitamin D deficiency, our data demonstrated that over 80% of female migrants from Arab and South Asian countries were vitamin D deficient. A retrospective study of the UAE population on 60,979 subjects originating from 136 different countries revealed that 78.9% of expatriates were suffering from hypovitaminosis, with more females being afflicted with severe deficiency [26]. Previous studies have shown lower prevalence among adults in developed countries, 24% in US [27], 37% in Canada [28], 32% in Australia [29], and 40% in the European countries [30]. Paradoxically, vitamin D deficiency of up to 80% was observed in Middle Eastern Arab countries despite the sunny weather [10,31]. Moreover, a systematic review and meta-analysis of the prevalence of vitamin D deficiency among the population of Africa on 21,474 individuals from 23 countries within the continent revealed the prevalence was higher than speculated at a rate of 34%; with women having remarkably lower 25(OH)D concentrations compared to men. It was concluded that being a female and living in urban areas in the northern and southern parts of Africa were associated with a higher risk of developing vitamin D deficiency [32]. The findings in the current study were in concordance with previous studies, and similarly documented a high prevalence of vitamin D deficiency among Arab female migrants originating from African and Middle Eastern countries. The reasons for vitamin D deficiency in Arab female migrants might include wearing conservative skin-concealing clothes, relatively high obesity, and low dietary vitamin D intake [9,10]. An additional factor might be related to dark skin complexion since the skin pigment melanin absorbs sunlight and dark skin color reduces the capacity of skin to synthesize vitamin D_3_ [33]. National examination surveys in the US indicated that over 80% of non-Hispanic black American adults, including men and women, had ≤20 ng/mL serum 25(OH)D concentrations [34,35].

Among South Asian females, 83.3% were vitamin D deficient in the present investigation. A recent systematic review and meta-analysis of vitamin D deficiency in Asia involving 472 studies on 746,564 individuals demonstrated that region and altitude were important correlates of vitamin D deficiency [36]. Data from different parts in India revealed a 70% prevalence of vitamin D deficiency among adult with a higher prevalence of up to 79% among females [37]. Studies conducted among South Asians in Australia, Canada, European countries, UK, and USA have found a prevalence of vitamin D deficiency in epidemic proportions compared to their native counterparts [34]. The proposed causes of vitamin D deficiency among South Asians in Western countries included low vitamin D intake, relatively high obesity prevalence, less exposure to sunlight, and wearing conservative clothes for cultural and religious reasons [38].

The low prevalence of vitamin D deficiency among Filipina migrants as compared to the South Asian and Arabs might be due to the lower prevalence of both obesity and physical inactivity [39]. However, additional confounders could also be involved including differences in dietary intake of vitamin D (as Filipinas highly consume oily fish) and dissimilarities in sartorial and lifestyle habits, which might entail more sun exposure and hence concomitant photosynthesis of vitamin D [40].

In our study, low physical activity, acculturation as measured by more than 5 years of residency in the UAE, and obesity were all independently associated with vitamin D deficiency. Physical activity was a significant independent predictor of vitamin D deficiency in several previous studies. Additionally, some researchers showed that serum 25(OH)D levels are affected by both physical activity and BMI in the context of obesity [41,42]. Pragmatically speaking, the positive impact of physical activity on vitamin D status could be attributed to metabolism upon energy disbursement with muscle contractions; however, when performed alfresco, it stimulates the conversion of dermal 7-dehydrocholesterol into vitamin D by solar UV-B rays [43]. The effect of physical activity could be complex, since physical inactivity is a well-established risk factor for overweight and obesity, which, in turn, could predispose to vitamin D deficiency through adipose sequestration and volumetric dilution of 25(OH)D [44]. Nevertheless, a study on older adults revealed a favorable correlation between physical activity and serum 25(OH)D levels over time regardless of exposure to sun [45]. In agreement with our results, numerous population-based investigations had demonstrated that obesity and reduced physical activity were independent determinants of vitamin D deficiency in South Asia [46], Philippines [40], Middle East [47], Europe, Australia, [29] and the US [35].

Obese females (BMI ≥ 30) in our study had a high prevalence of vitamin D deficiency. It is worth mentioning that obesity has been well established as a risk factor for vitamin D deficiency. Several studies have demonstrated a close association between vitamin D deficiency and obesity among the Asian and Arab adult population [48]. Data from the World Health Organization (WHO) reveal that the UAE currently ranks fifth in the world in obesity, at a prevalence rate of 36% (33% for males and 39% for females) [49].

The rapid socioeconomic transition in MENA countries has resulted in increased urbanization and drastic lifestyle changes and has also manifested in sedentary and unhealthy dietary practices. These factors combined with the growing fast-food industry has significantly impacted the prevalence of obesity among this population. Vitamin D deficiency and the rapid increase in the prevalence of obesity are both considered important public health issues contributing to morbidity and mortality in the UAE [50].

A series of evidence support the fact that obesity might be driving low serum 25(OH)D concentrations due to decreased bioavailability of vitamin D through sequestration within body fat and volumetric dilution [36,42]. It has been proposed that dietary calcium and vitamin D status might play a role in weight and fat regulation [51]. According to this hypothesis, lower calcium or vitamin D results in increased parathyroid hormone (PTH), which in turn decreases 25(OH)D and increases intracellular calcium into adipocytes, consequently inhibiting lipolysis and stimulating lipogenesis [52]. Furthermore, there are profound indications for an association between vitamin D levels and obesity, and that serum level of 25(OH)D are reduced in obese subjects in adults [53].

The data we analyzed highlighted that length of stay of greater than 5 years in the UAE was a strong predictor of vitamin D deficiency among female immigrants. This parameter is often used as an indicator of adjustment to a new culture acculturation. The relation between acculturation and immigrants’ health is quite complex given the diversity of cultures and how temporal adaptation might affect health outcomes by embracing new habits that could act as risk factors for diseases, such asreduced physical activity and obesity [54]. A cross-sectional study in Canada that examined the prevalence of vitamin D deficiency among first generation immigrants (n = 11,579) concluded that the length of time lived in Canada, lifestyle, and ethnicity were important correlates. Overall, immigrants had significantly lower levels of 25(OH)D compared to native-born Canadians with Arabs and Southeast Asians in comparison to other white ethnic groups having the highest levels of vitamin D deficiency [55].

Immigration studies exploring the impact of acculturation on vitamin D status have demonstrated a significant positive correlation with the length of stay and serum 25(OH)D among immigrants in Canada [56]. This was in contrast with our findings and could be explained by the fact that integration into a new modernized life in the UAE with a thriving fast-food industry and more sedentary life could be the major culprit for the negative aspect of acculturation [55]. Importantly, food fortification with vitamin D in the UAE, unlike Canada, is not strictly implemented and this might also explain the conflicting results we obtained [10,27].

However, our results are in concordance with reported findings from numerous other studies which examined the relationship between culture and health in the context of acculturation on the health of Asian immigrants in the US, Australia, and Europe [55]. A recent review confirmed that vitamin D deficiency was at epidemic levels among South Asian immigrants living in Western countries mainly due to low dietary intake of vitamin D, high obesity, avoidance of sun exposure, and having covered sartorial dress style either due to cultural or religious reasons [38,57]. Moreover, investigators concluded that being a female, living in an urbanized setting, and limiting physical activity in Southeast Asia were strongly correlated with a low vitamin D status. In addition, religious, behavioral practices, and nutritional dissimilarities between Muslims and non-Muslims contributed to the remarkable differences in vitamin D status among these two subgroups of the same population in Thailand, with non-Muslims having 10 nmol/L higher levels of 25(OH)D [56]. The effect of clothing preference on vitamin D status of females has been well described by other researchers [58].

Vitamin D deficiency is a global public health crisis to which immigrants are predominantly susceptible especially with sub-populations that have darker skin and who move to the Western regions that have high latitudes [59]. In general, however, the vitamin D status of northern African countries was similar to populations in the Middle East, most likely due to similar environmental and behavioral styles [9]. In comparison to other ethnicities, immigrants from the African descent who settled in temperate areas in the US and Europe had a higher prevalence of vitamin D deficiency [60,61]. Moreover, national surveys in Europe reported that approximately 40% of these sub-populations had 25(OH)D concentrations below the sufficiency cutoff [62]. This pattern had been ascribed to the reduced adaptability of their darker skin color for vitamin D photosynthesis in regions that are not as sunny as Africa. Concurrently, a remarkable prevalence of 82.1% for vitamin D deficiency was documented among African American in the US in comparison to a nationwide mean value of 41.9% [60]. Moreover, a decrease in 25(OH)D concentrations was consistently reported with more northern latitudes and the duration of time since moving from Africa [59]. In a large sample (*n* = 12,346) of native Emirati population, a high proportion (84%) of native Emirati females had vitamin D deficiency (<50 nmol/L) [63]. The predisposing factors for the vitamin D deficiency were high BMI, central obesity, high blood pressure, high cholesterol, and impaired blood glucose levels [64].

## 5. Strengths and Limitations

This study is the first to examine the prevalence of vitamin D deficiency among an under-studied and vulnerable subpopulation of female migrants in the UAE. Low vitamin D status along with other biomarkers among this subpopulation could signify health deterioration and the need for action. Our findings have valuable global health implications regarding the physical wellbeing of foreign immigrants and could inform future studies aiming to respond to chronic and infectious diseases including COVID-19 [63]. Moreover, the data obtained was for immigrants from different countries and could present a unique understanding to the cultural diversity and its intricate relation to immigrants’ health within the UAE.

Despite the strength of the study, there are several limitations that should be acknowledged. The cross-sectional nature of design does not confirm causation of vitamin D deficiency, and hence findings remain correlational. Serum 25(OH)D concentrations are frequently used as clinical indicators for the assessment of vitamin D status, which is determined by sun exposure, dietary intake, and supplementation. In this research, such data was not available for analysis. However, the impact of seasonal variation in this study was minimal since the research was conducted in the summer, during which the contribution to vitamin D status by sun exposure is often restricted, since residents of the UAE avoid the sun and heat during this very hot season and spend their time indoor in cool air-conditioned settings [27,51]. We did not collect information on dietary habits, and/or supplement use, Fitzpatrick skin type, or tanning/outdoor habits. Vitamin D status is affected by diseases pertaining to impaired digestion and malabsorption like Crohn’s and Celiac diseases; we did not collect this data in our study.

Examining differences in dietary habits, as well as behavioural practices towards sun exposure among the different subpopulations of female immigrants, are worth further investigation. The attitude and practices of the participants towards sun exposure were not explored but might have deterred them from securing an adequate vitamin D status. Whiter skin complexion had been associated with beauty, attractiveness, higher socio-economic advantages, and prestige among females from some populations, particularly in South Asia and Africa [65,66,67,68]. Lastly, genetic factors are also an important element to consider in such research owing to the contribution of genetic variants within vitamin D metabolism to the vitamin D status [68].

## 6. Conclusions

In conclusion, vitamin D deficiency is highly prevalent among female immigrant workers in the UAE. A prevention strategy including Vitamin D supplementation and educating this vulnerable subpopulation is urgently needed. Currently, the vitamin D recommendations for the UAE do not contain any specific guidelines for the immigrant subpopulations. Until food fortification with vitamin D becomes effective in the UAE, sun exposure and the use of supplements remain the most significant determinants of vitamin D status for the UAE population. Our study might have important connotations for introducing culturally acceptable approaches of increasing skin exposure to the sun and a subpopulation specific vitamin D dietary intake.

## Figures and Tables

**Table 1:** Characteristics of the female migrant participants living in Al Ain, UAE.

Variable		Filipinas		Arab		South Asian	
	*N*	*n*	%	*n*	%	*n*	%
All	553	290	52.4	136	24.6	127	23.0
Age, (years)							
18–30	186	123	(43.0)	43	(31.6)	55	(43.7)
31–40	171	119	(41.6)	45	(33.3)	39	(39.9)
≥41	191	44	(15.4)	47	(34.8)	32	(25.4)
Education of the participant							
No formal schooling	39	15	(5.2)	15	(11.2)	9	(7.3)
Up to secondary	203	124	(43.2)	40	(29.8)	59	(31.4)
College or higher	303	148	(51.6)	79	(59.0)	76	(61.3)
Marital status							
Unmarried	194	137	(49.6)	34	(25.6)	23	(18.6)
Married	306	118	(42.8)	89	(66.9)	99	(79.8)
Divorced, or widowed	33	21	(7.6)	10	(7.5)	2	(1.6)
Occupation							
Housemaid	192	176	63.6	8	6.2	8	6.8
Housewife	122	4	1.4	64	49.6	54	45.8
Driver	10	10	3.6	0	0	0	0.0
Cook	10	10	3.6	0	0	0	0.0
Administrator, supervisor	35	21	7.6	7	5.4	7	5.9
Teacher	36	5	1.8	16	12.4	15	12.7
Health care worker	55	23	8.3	13	10.1	19	16.1
Other	64	28	10.1	21	16.3	15	12.7
Monthly income, AED (1 USD = 3.6 AED)							
Lowest (812.6)	133	125	(52.5)	5	(13.2)	3	(6.5)
Middle (1365.8)	85	72	(30.3)	5	(13.2)	8	(17.4)
Highest (7422.8)	104	41	(17.2)	28	(73.6)	35	(76.1)
Residence in home country, *n* (%)							
Urban	263	129	(50.8)	54	(43.2)	80	(67.2)
Rural	235	125	(49.2)	71	(56.8)	39	(32.8)
Duration of residence in UAE							
<1 year	185	133	(53.0)	27	(22.9)	25	(22.9)
1 to 5 years	208	106	(42.2)	43	(36.4)	59	(54.1)
≥5 years	85	12	(4.8)	48	(40.7)	25	(23.0)
Moderate or vigorous physical activity							
Yes	161	133	(45.9)	15	(11.0)	13	(10.2)
No	391	157	(54.1)	121	89.0)	114	(89.8)
Body mass index categories							
<25.0	294	187	(64.5)	44	(32.4)	63	(49.6)
25–30	166	83	(28.6)	41	(30.1	42	(33.1)
≥30.0	93	20	(6.9)	51	(37.5)	22	(17.3)
Cigarette smoking, currently							
No	514	268	(92.4)	122	(89.7)	124	(97.6)
Yes	39	22	(7.6)	14	(10.3)	3	(2.4)
Alcohol consumption							
No	448	193	(66.6)	135	(99.3)	120	(94.5)
Yes	105	97	(33.4)	1	(0.7)	7	(5.5)
Hemoglobin A1c level							
<5.7%	151	52	(91.2)	64	(71.1)	35	(53.9)
5.7–6.4%	38	4	(7.0)	15	(16.7)	19	(29.2)
≥6.5%	23	1	(1.8)	11	(12.2)	11	(16.9)
Blood pressure, mm Hg	363	188	(64.8)	93	(68.4)	82	(64.6)
<140/90	190	102	(35.2)	43	(31.6)	45	(35.4)
≥140/90 or on hypertension medication							
Levels of the serum 25(OH)D concentrations (ng/mL)							
Mean (ng/mL, (± SD)	20 (±12)	30	(±11)	14	(±10)	15	(±9)
Serum 25(OH)D concentrations by category							
>20 ng/mL (50 nmol/L), *n* (%)	71 (33.3)	48	(84.2)	12	(13.3)	11	(16.7)
≤20 ng/mL (50 nmol/L)	142 (66.7)	9	(15.8)	78	(86.7)	55	(83.3)

Data are presented as *N* and *n* (%); data presented as mean ± SD.

**Table 2:** Distribution of inadequate 25(OH)D levels (ng/mL) among the female migrant participants according to sociodemographic, lifestyle, and clinical characteristics.

Variable		25(OH)D Levels (ng/mL)					
					<20 ng/mL)		
	*N*	Mean	(±SD)	*p*	%	(95% CI)	*p*
All	213	19	(11)		66.7	(60.0–72.7)	
Age, (years)							
18–34	113	18	(12)	0.37	68.1	(58.9–76.1)	0.38
35–44	57	20	(14)		59.6	(47.1–72.2)	
≥45	43	18	(11)		72.1	(55.8–83.2)	
Nationality							
Filipinas	57	30	(12)	<0.001	15.8	(8.3–27.8)	<0.001
Arab	90	14	(10)		86.7	(77.8–92.3)	
South Asian	66	15	(9)		83.3	(72.2–90.6)	
Education of the participant							
No formal schooling	18	17	(15)	0.02	77.8	(52.6–91.7)	<0.001
Up to secondary	91	22	(13)		49.4	(39.2–59.7)	
College or higher	101	17	(10)		79.2	(70.1–86.1)	
Marital status							
Unmarried	69	21	(13)	0.26	56.5	(44.5–67.58	0.06
Married	129	18	(11)		72.9	(64.5–79.8)	
Divorced, or widowed	10	19	(12)		60.0	(28.2–85.1)	
Occupation							
Housemaid	64	29	(12)	<0.001	21.9	(13.3–33.8)	<0.001
Housewife	71	14	(9)		88.7	(78.9–94.3)	
Driver	8	12	(7)		87.5	(31.9–98.1)	
Administrator, supervisor	9	14	(5)		77.8	(39.6–94.9)	
Teacher	21	15	(10)		80.9	(58.0–92.9)	
Health care worker	18	18	(8)		77.8	(52.6–91.7)	
Other	22	14	(13)		90.9	(69.1–97.8)	
Monthly income, Dirham (AED)							
(US dollar = 3.7 AED)							
Lowest (801.2)	42	29	12	<0.001	23.8	(13.1–39.3)	<0.001
Middle (1386.3)	19	24	13		36.8	(18.1–60.6)	
Highest (8397.5)	40	17	13		80.0	(64.4–89.8)	
Residence, n (%)							
Urban	84	19	13	0.74	65.5	(54.6–74.9)	0.57
Rural	114	19	12		69.3	(60.1–77.1)	
Duration of residence in UAE							
<1 year	62	22	14	0.005	56.4	(43.8–68.2)	<0.001
1 to 5 years	72	18	11		65.3	(53.5–75.4)	
≥5 years	49	14	14		91.8	(79.69–96.9)	
Physical activity (mod/vigorous.)							
Yes	52	25	13	<0.001	42.3	(29.5–56.2)	<0.001
No	161	17	11		75.5	(67.2–80.7)	
Body mass index categories				0.002			
<25.0	94	21	13		57.4	(47.62–67.1)	0.007
25–30	74	19	12		67.6	(55.9–77.3)	
≥30.0	45	14	8		84.4	(70.5–92.4)	
Cigarette smoking, currently							
No	199	19	12	0.99	65.3	(17.1–20.5)	0.12
Yes	14	19	16		85.7	(9.5–28.0)	
Alcohol use							
No	197	18	12	<0.001	71.1	(64.3–77.0)	<0.001
Yes	16	32	13		12.5	(2.9–39.9)	
Hemoglobin A1c level							
<5.7%	151	20	13	0.29	62.2	(54.2–69.7)	0.11
5.7–6.4%	38	16	8		79.9	(62.8–89.2)	
≥6.5%	23	19	13		73.9	(52.1.3–88.1)	
Blood pressure, mm Hg							
<140/90	139	18	13	0.45	70.5	(62.3–77.5)	0.10
≥140/90 or on hypertension medication	74	20	11		59.5	(47.8–70.1)	

Data are presented as frequencies (%) and mean ± SD and odds ratio (OR) (95% CI); *p* < 0.05 considered significant (shown in boldface).

**Table 3:** Multivariate logistic regression analysis for vitamin D deficiency correlates among the female migrant participants.

Determinants	Adjusted		
	OR	95% CI	*p*
Age in years	0.97	(0.92–1.01)	0.18
Nationality			
Filipinas	0.06	(0.01–0.42)	0.004
Arab	Reference		
South Asian	0.79	(0.32–1.98)	0.62
Education			
No formal education	Reference		
Up to secondary	0.24	(0.05–1.04)	0.06
College or higher	0.66	0.15–2.89)	0.59
Duration of residence in UAE			
≤1 year	Reference		
>1 to 5 years	0.78	(0.33–1.83)	0.67
>5 years	4.65	(1.31–16.53)	0.02
Body mass index categories			
BMI			
≤24.99	Reference		
25–29.99	2.21	(0.92–5.30)	0.08
≥30.0	3.56	(1.04–12.20)	0.04
Low level of physical activity *			
No	Reference		
Yes	4.59	(1.98–10.63)	<0.001

* Self-reported (IPAQ questionnaire) moderate activity of 30 min on five days or vigorous intensity. Physical activity for a minimum of 20 min on three days each week. Data are presented as frequencies (%) and odds ratio (OR) (95% CI); *p* < 0.05 considered significant (shown in boldface).

## Data Availability

The data presented in this study are available on request from the corresponding author.
